# ﻿A new genus of Assamiidae (Opiliones, Grassatores) from Xizang, China

**DOI:** 10.3897/zookeys.1215.132189

**Published:** 2024-10-11

**Authors:** Xiaoru Qi, Adriano B. Kury, Chao Zhang

**Affiliations:** 1 Key Laboratory of Zoological Systematics and Application, College of Life Sciences, Hebei University, Baoding, Hebei 071002, China; 2 Hebei Basic Science Center for Biotic Interaction, Hebei University, Baoding, Hebei 071002, China; 3 Departamento de Invertebrados, Museu Nacional/Universidade Federal do Rio de Janeiro (UFRJ), Quinta da Boa Vista, São Cristóvão, 20.940-040, Rio de Janeiro, RJ, Brazil

**Keywords:** Arachnida, Eastern Himalayas, genitalia, harvestmen, taxonomy

## Abstract

Knowledge of the family Assamiidae in the Eastern Himalayas is primarily concentrated from Nepal, while Bhutan, Xizang, and northeastern India remain much less studied. Herein, a new genus, *Linzhiassamia***gen. nov.**, is described from Xizang, China, along with two new species, *Linzhiassamiamedogensis***sp. nov.** and *Linzhiassamiazayuensis***sp. nov.** These represents the first records of the family Assamiidae from Xizang. A comparative analysis with other continental Asian Assamiidae is undertaken, with a focus on genital morphology. Potential closest relatives are identified, including the genus *Dhaulagirius* Martens, 1977 from Nepal.

## ﻿Introduction

The Paleotropical family Assamiidae encompasses more than 450 valid species (Kury et al. 2023). In stark contrast to families that have “prospered from renewed interest and revitalized sampling efforts,” assamiids are ensnared in a “labyrinthine taxonomy” (Palmieri et al. 2023), a legacy of the Roewerian system. Currently, Assamiidae is divided into 13 mostly Roewerian subfamilies, the monophyly of which is not clearly supported, and some of which exhibit suspiciously disjunct geographic distributions. Palmieri et al. (2023) utilized a 10-locus Sanger dataset for a limited number of terminals and found that, as is common in most harvestmen, the phylogeny of Assamiidae is heavily dependent on geography and clades do not correspond to traditional subfamilies. However, the generic and subfamilial boundaries within the family remain entirely unclear.

Modern descriptions of assamiid species are sparse and even combined, they fail to encompass their entire taxonomic and geographic range. Specifically, Kauri (1961, 1985), Bauer and Prieto (2009), Santos and Prieto (2010), Lotz (2011), and Martens (2022) have contributed to the understanding of the Afrotropical clade sensu Palmieri et al. Suzuki (1969a, 1969b), Zhang and Zhang (2015), Zhang et al. (2010) and Zhao et al. (2023) have contributed to knowledge of assamiids from Indochina (including Yunnan) and Borneo. Finally, Martens (1977, 2021) has contributed detailed descriptions from Nepal. Notably, there are no modern descriptions from the Indian subcontinent, and there are no records of the family from Xizang.

In this article, two new species from Xizang are described. Unable to find an existing genus that matches their characters, we have erected the new genus *Linzhiassamia* gen. nov. Additionally, we describe the intraspecific variation of *L.zayuensis* sp. nov., providing detailed morphological descriptions, observations of genitalia, and discussions on the taxonomic position of the new species.

### ﻿Taxonomic background of the Assamiidae

Assamiidae was initially a monogeneric family for *Assamia* Sørensen, 1884 from the Indian highlands. Thorell (1889) added *Maracandus* and three new genera, then later added two more South-East Asian genera (Thorell 1890, 1891). Sørensen (1896) described four new African genera and merged Dampetroidae into Assamioidæ. Loman (1902) confirmed the inclusion of Dampetridae, added Samoidae to the synonymy, and described eight new genera. Roewer (1912) misspelled the family name, restored Samoidae as a subfamily of Phalangodidae, included two former Ethiopian Epedanidae, removed *Conomma* and *Mitraceras*, and distinguished three subfamilies: Assamiinae, Dampetrinae, and Trionyxellinae. Roewer (1923, 1927) added more genera and species. In 1935, Roewer created 14 new subfamilies and numerous new genera and species. Roewer (1940) added more genera and species without changing the suprageneric classification. Mello-Leitão (1949) separated the pseudonychiate subfamilies into the new family Trionyxellidae.

Kauri (1985: 42) introduced the subfamily Irumuinae, comprising specialized subterranean species that inhabit forest soil and humus layers. In contrast, Staręga (1992: 273) opted to omit subfamilies from his catalog of genera, as he did for other families, effectively synonymizing two subfamilies by merging their type genera into other subfamilies: Tsadseinae Roewer, 1935 into Erecinae Roewer, 1935, and Harsadiinae Roewer, 1935 into Sidaminae Roewer, 1935. Kury (2007) succinctly summarized the taxonomy of the family, criticizing the current subdivisions as unsatisfactory and artificial. More recently, Martens (2022) described a new subfamily from the Ethiopian highlands, characterized by unusual pedipalp dimorphism, although the male genitalia conform to the family’s basic morphology.

Recent trends in taxonomy have seen a dismissal of subfamilies that are not clades. For instance, Palmieri et al. (2023: 6) highlighted the non-monophyly of certain subfamilies, thereby questioning the utility of several subfamily definitions. Similarly, Klementz and Sharma (2023: 34) referred to the “now taxonomically defunct Polycoryphinae or Mysoreinae subfamilies.” However, this approach seems to reflect our current challenges in developing robust morphological diagnoses, which stem from a lack of comprehensive taxonomic groundwork. Discarding these subfamilies should be seen as a transition towards a more accurate and phylogenetically informed taxonomy, particularly considering the nearly 500 species within the Assamiidae that require intermediate taxa. Our focus should be on reforming and refining these subfamilies by leveraging both existing and new morphological data to enhance their utility and accuracy.

## ﻿Materials and methods

### ﻿Specimen preparation and examination

The specimens were preserved in 75% ethanol, examined under a Leica M205A stereomicroscope, and the overall drawings were made using a drawing tube-equipped Leica M205A stereomicroscope, while the detailed drawings were created using Inscape v. 1.3. Photographs were taken using a Leica M205A stereomicroscope, equipped with a DFC 450 CCD. The male genitalia were initially placed in hot lactic acid (40–50 °C) for about 1–2 min, then transferred to distilled water; the movable parts of the glans will mostly expand within 1 min (Schwendinger and Martens 2002).

### ﻿Terminology

The terminology of genital structures follows Martens (1986) and Macías-Ordóñez et al. (2010), and the macrosetae terminology of male genitalia follows Kury and Machado (2021). Terminology for the outline of the dorsal scutum follows Kury and Medrano (2016). Type specimens of the new species are deposited in the Museum of Hebei University, Baoding, China (**MHBU**). All measurements are given in millimeters. The following abbreviations are used in the text: Pb pars basalis; Pd pars distalis.

### ﻿Comparisons

In the diagnosis given here, *Linzhiassamia* sp. nov. is assessed in relation to genera that possess some features in common, such as the Nepalese highlanders *Dhaulagirius* Martens, 1977; *Micrassamula* Martens, 1977; *Nepalsia* Martens, 1977; and *Nepalsioides* Martens, 1977, as well as *Nilgirius* Roewer, 1915, which occurs in Yunnan and India. It is also compared with *Paktongius* Suzuki, 1969, from the lowlands of Laos, Malaysia, and Thailand.

### ﻿Taxonomy

#### ﻿ Linzhiassamia gen. nov.

Taxon classificationAnimaliaOpilionesAssamiidae

92A8F083-F482-5665-9936-19C090577F8D

https://zoobank.org/909654AF-C499-4D06-A842-555ECAFC699F

##### Included species.

*Linzhiassamiamedogensis* sp. nov. (type species) and *Linzhiassamiazayuensis* sp. nov.

##### Etymology.

The genus name is based on 林芝 (Linzhi), an alternative name for Nyingchi. This is associated with the pre-existing genus name *Assamia*. The gender is feminine.

##### Diagnosis.

*Linzhiassamia* is similar to *Paktongius* for the sexually dimorphic coxa IV, which in males may reach areas III, IV or even V of the dorsal scutum, while in females it is much shorter, reaching area II, and not projected laterally (*Nilgirius* and the Nepalese genera treated here, all possess coxa IV monomorphic).

*Linzhiassamia* is similar to *Dhaulagirius* due to the sexually dimorphic chelicerae; however, in *Dhaulagirius*, the cheliceral hand is much more exaggeratedly developed. In the other genera compared here, the chelicerae are monomorphic.

*Linzhiassamia* is similar to *Nilgirius* and *Paktongius* for having a “pseudonychium” (tarsal process) in the tarsi of legs III and IV.

*Linzhiassamia* is similar to *Dhaulagirius*, *Micrassamula*, and *Nepalsia* for lacking a sharp annular joint-like constriction in the distal third of the truncus penis, between the pars basalis and pars distalis (in contrast to *Nepalsioides* and *Nilgirius*).

*Linzhiassamia* is similar to *Dhaulagirius*, *Nepalsia*, and *Nepalsioides* for having MS A organized in a triangle (in contrast to *Micrassamula*, which has MS A extended into a line, and *Nilgirius*, which has a ring-girdle formed by macrosetae D1, A1–A3, and B1).

*Linzhiassamia* is similar to *Dhaulagirius*, *Nepalsia*, and *Nilgirius* for not having a wide dorsal concavity, giving the distal truncus the aspect of an ice-cream scoop (in contrast to *Micrassamula* and *Nepalsioides*).

*Linzhiassamia* is similar to *Nepalsia* and *Nilgirius* for having all macrosetae concentrated distally (in contrast to *Dhaulagirius*, *Micrassamula*, and *Nepalsioides*).

*Linzhiassamia* is similar to *Dhaulagirius* for having the distal part of the truncus with constrictions, flaring to form a pyriform structure (in contrast to *Micrassamula*, *Nepalsia*, *Nepalsioides*, and *Nilgirius*, which have more or less continuous widening of the truncus, getting rounded apically).

#### ﻿ Linzhiassamia medogensis sp. nov.

Taxon classificationAnimaliaOpilionesAssamiidae

0540D128-7456-5EF9-B54D-87118B5DD9EE

https://zoobank.org/F56C8DB5-9ADA-4DDD-9E87-04D420941FC1

Figs 1–6 , 7–12 , 13–19 , 20–25

##### Type material.

***Holotype*** • male (MHBU-Opi-24ZC011501): China: Xizang, Nyingchi, Medog County, 29°33'N, 95°33'E, alt. 1116 m, 22 May 2019, H. Wang, L.Y. Wang leg. ***Paratypes***: • one male (MHBU-Opi-24ZC011502) and two females (MHBU-Opi-24ZC011503-04), same collecting data as holotype • one male (MHBU-Opi-24ZC011601): China: Xizang, Nyingchi, Medog County, 29°33'N, 95°33'E, alt. 1116 m, 23 May 2019, H. Wang leg.

##### Diagnosis.

Distal section of penis (pars distalis) markedly enlarged: ventral plate nearly triangle and frontal rim with median crevice (Figs 13, 15), convex in dorsal view and concave in ventral view (Figs 14, 16); The pars basalis and pars distalis of the penis are connected by joints (Figs 13–17). Glans partially sunken into a dorsally depressed portion of pars distalis of penis, its tip slightly extending the distal margin (Fig. 14) of the ventral plate. Opisthosomal region of scutum with abundant setiferous tubercles. Ocularium without spines, but with small, scattered tubercles. Pedipalpal femur ventrally with a row of six or seven setiferous tubercles.

##### Etymology.

The species name originates from the specimen collection site: Medog County, Nyingchi, Xizang.

##### Description.

**Figures 1–6.**
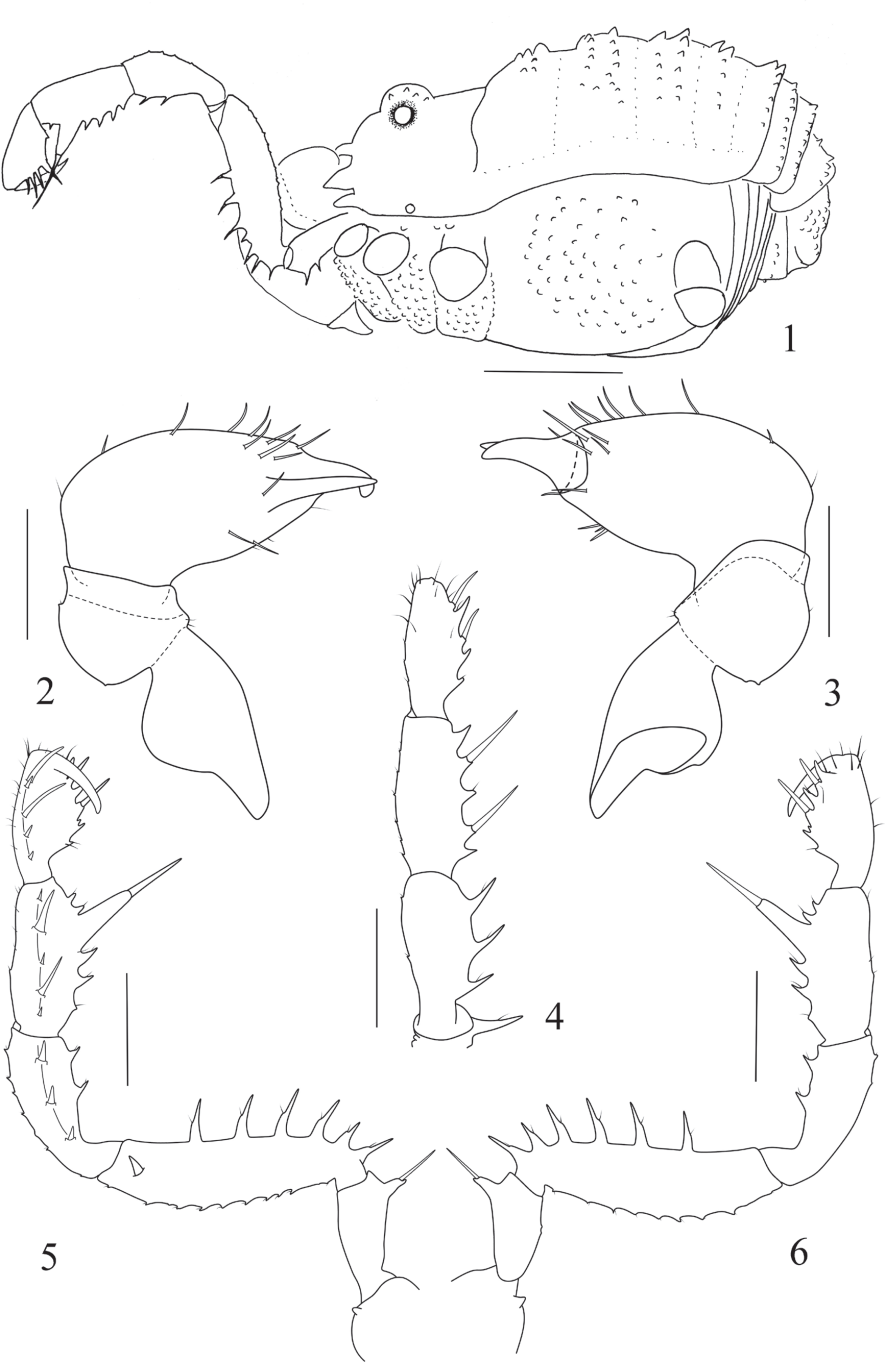
*Linzhiassamiamedogensis* sp. nov., male (**2–6** holotype), male (**1** paratype) **1** male body, lateral view **2** left chelicera of male, ental view **3** left chelicera of male, ectal view **4** left pedipalp of male, dorsal view **5** left pedipalp of male, ental view **6** left pedipalp of male, ectal view. Scale bars: 1 mm (**1**); 0.5 mm (**2–6**).

**Figures 7–12.**
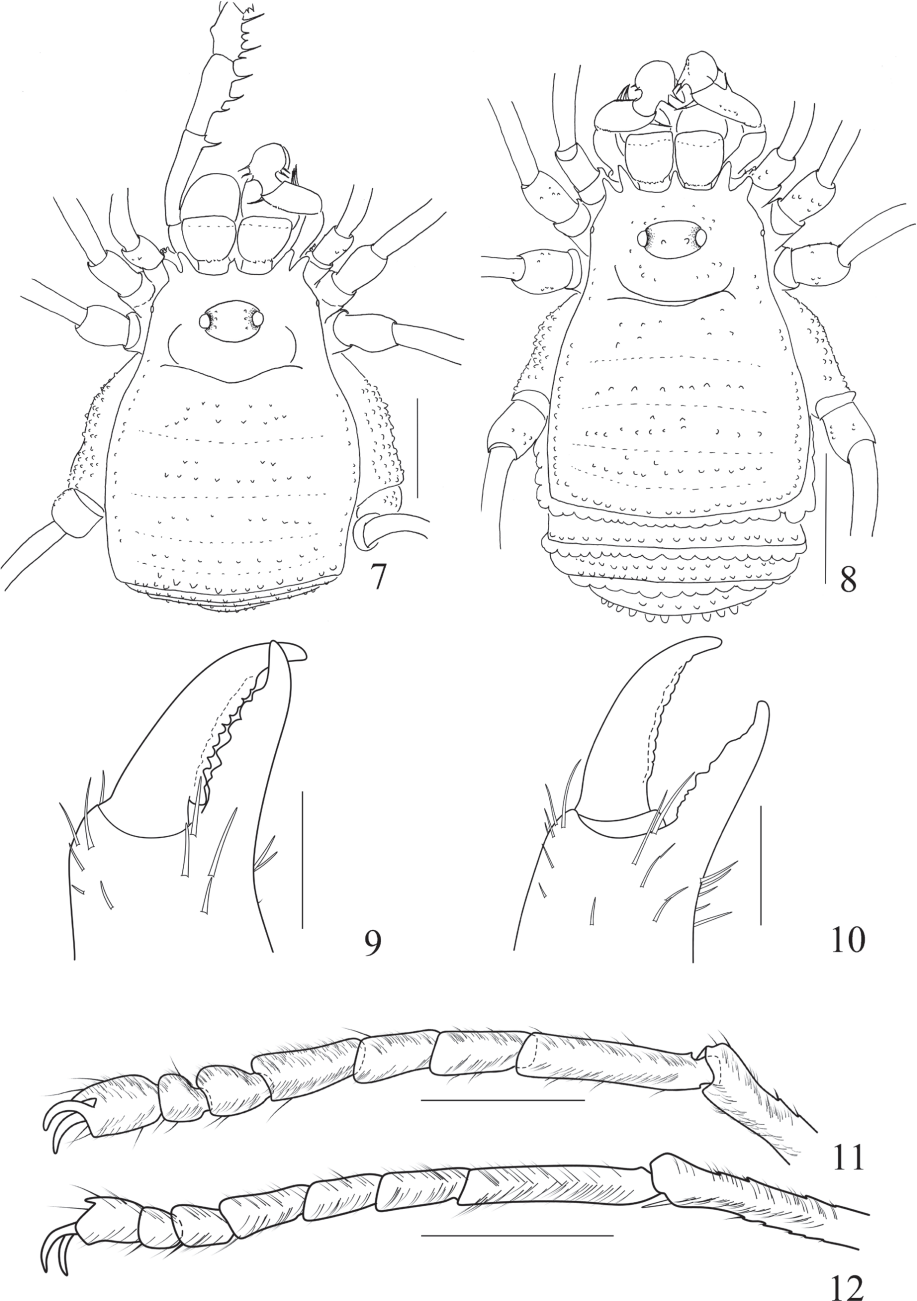
*Linzhiassamiamedogensis* sp. nov., male (**9, 11** holotype), male (**7** paratype), female (**8, 10, 12** paratype) **7** male body, dorsal view **8** female body, dorsal view **9** left cheliceral fingers of male, frontal view **10** left cheliceral fingers of female, frontal view **11** right tarsal claw IV of male, lateral view **12** right tarsal claw IV of female, lateral view. Scale bars: 1 mm (**7, 8**); 0.5 mm (**11–12**); 0.25 mm (**9–10**).

**Male** (holotype and paratype). Habitus as in Figs 1, 7, 20–22. Coloration (Figs 20–22): entire body dorsally rusty yellow with brown patches; median area of prosoma with dark brown reticulations before and behind the interocular mound; around the ocularium dark brown patches; both lateral ridges of the prosomal and opisthosomal scuta with dark brown stripes; opisthosomal areas I–IV with dark brown, and the central region being lighter than the surrounding areas; there are transverse paler interspaces between areas I–III; area V and free tergites each with a transverse dark band; venter concolorous with the dorsum; chelicerae, pedipalps and legs rusty yellow, reticulated with light to dark brown.

**Figures 13–19.**
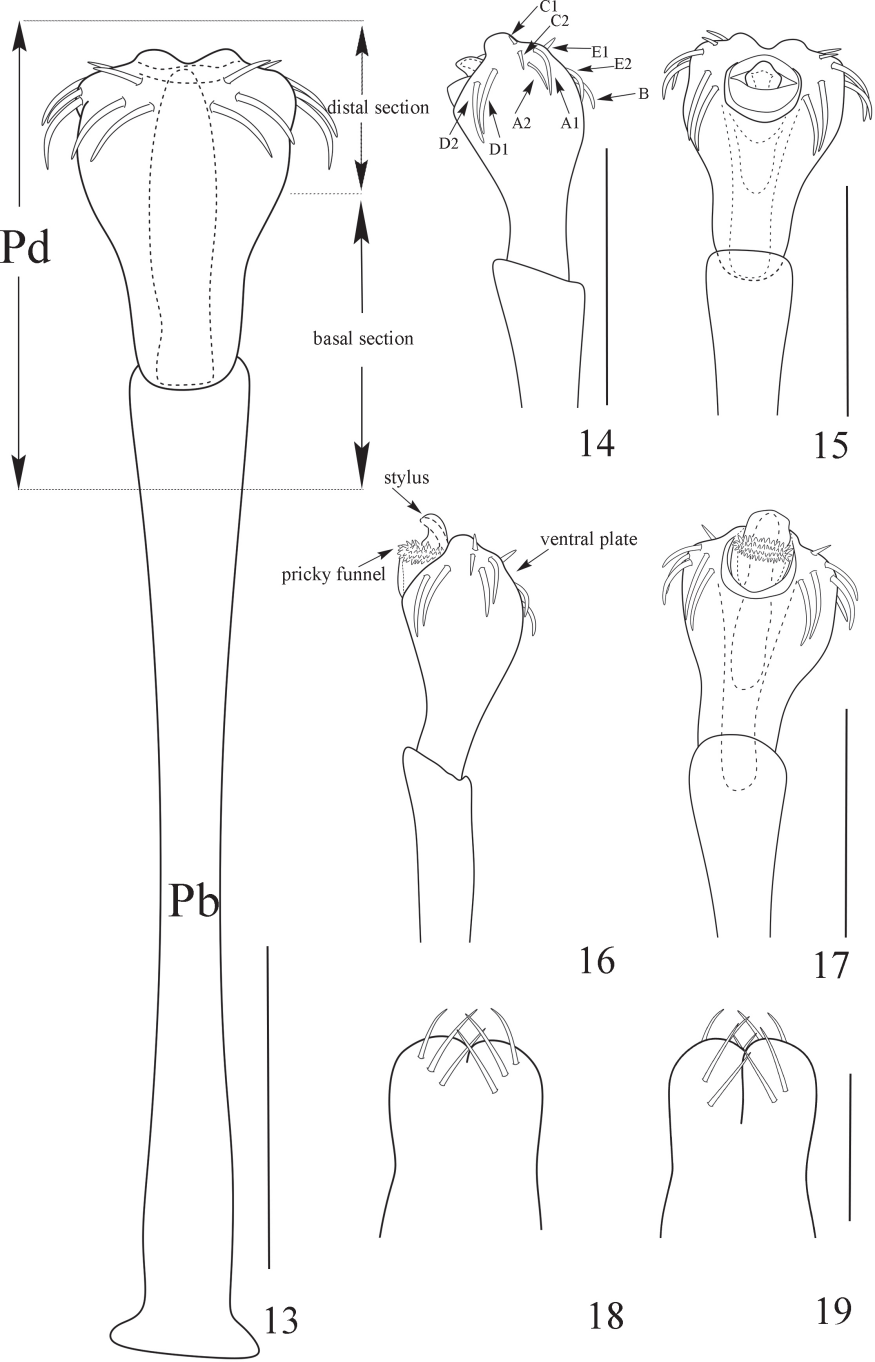
*Linzhiassamiamedogensis* sp. nov., genitalia of male holotype (**13–17**) and female paratype (**18–19**) **13** penis, ventral view **14** distal part of penis, lateral view **15** same, dorsal view **16** distal part of penis (expanded), lateral view **17** same, dorsal view **18** ovipositor, dorsal view **19** same, ventral view. Pb, pars basalis, Pd pars distalis. Scale bars: 0.25 mm.

**Figures 20–25.**
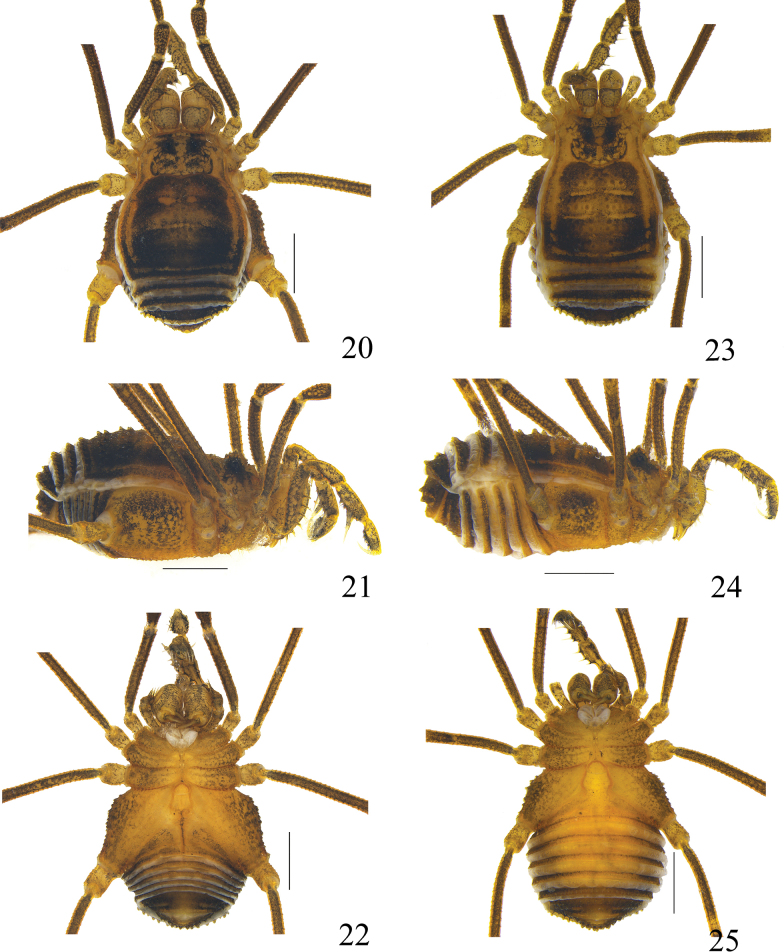
Photographs of male (**20–22** holotype) and female (**23–25** paratype) of *Linzhiassamiamedogensis* sp. nov. **20, 23** body and parts of appendages, dorsal view **21, 24** body and parts of appendages, lateral view **22, 25** body and parts of appendages, ventral view. Scale bars: 1 mm.

***Dorsum*** (Figs 7, 20). Dorsal scutum pyriform in shape, widest portion of body at scutal area II. Anterior margin of carapace with two spines at the lateral portion and a single median spine, the middle one is the smallest. Entire prosoma covered with small tubercles; anterior margin of prosoma with a row of small tubercles at the lateral portion. Ocularium oval, removed from the anterior border of scutum by 0.13 mm, and scattered with small tubercles. Opisthosomal region of scutum with five areas. Opisthosomal areas I–IV are adorned with abundant seta-tipped tubercles and a longitudinal row of similar tubercles on the lateral margins. Area V and all free tergites with a transverse row of seta-tipped tubercles.

Venter (Fig. 22). Surface of all coxae tuberculated. Coxa I with a row of four tubercles prolaterally, and two rows of tubercles on the surface. Coxa II with a row of marginal tubercles on the prolateral surface, and disto-dorsally with an enlarged tubercle. Coxa III with prolateral and retrolateral rows of tubercles. Coxa IV larger than others, prolaterally with many scattered tubercles, Genital operculum with many hair-tipped granules. Free sternites with a row of minute tubercles, each with setae on top. Spiracles concealed.

***Chelicera*** (Figs 2–3, 9). Basichelicerite elongate, dorsally with a slight bulla, without prominent armaments. Cheliceral hand unarmed, with sparse hairs only. Fingers relatively short, inner edges toothed as illustrated (Fig. 9): moveable finger with 11 teeth, the proximal one enlarged; fixed finger with six teeth, the proximal one diminished.

***Pedipalpus*** (Figs 4–6). Coxa dorsally with one small tubercle. Trochanter ventrally with one long distal setiferous tubercle. Femur compressed laterally, widest at the middle of its length, ventrally with a row of 6 homogeneous setiferous tubercles; dorsally with a row of twelve low conical tubercles along the entire length; on the medial distal side with one setiferous tubercle. Patella with three ventromesal setiferous tubercles, and two ventroectal setiferous tubercles, dorsally with a row of six low conical tubercles along the entire length. Tibia ventromesally with two enlarged and three small setiferous tubercles; and ventroectally with one fairly enlarged and five setiferous tubercles. Tarsus with sparse hairs, ventromesally with two slightly enlarged and three small setiferous tubercle, and ventroectally with two slightly enlarged and five small setiferous tubercles. Tarsal claw slightly curved, shorter than tarsus.

***Legs*.** Slender and elongated. Trochanters I–IV with small, hair-tipped granules on the ventral surface. All femora with hair-tipped granules, femora III and IV curved. Tarsi III–IV with a pseudonychium and two bare claws (Fig. 11). Tarsal formula (I–IV): 5(2)/11–14(3)/6/7. Distitarsus I two-jointed and II three-jointed. The remaining leg segments with hair-tipped granules.

***Penis*** (Figs 13–17). Truncus (pars basalis) slender, sides nearly parallel, then slightly enlarged (Fig. 13) and curved (Figs 14, 16) towards distal end. Distal portion of penis (pars distalis) markedly enlarged: ventral plate nearly triangle and frontal rim with median crevice (Figs 13–15), convex in dorsal view and concave in ventral view (Figs 14, 16); pars basalis and pars distalis of the penis connected by joints. Glans partially sunken into a dorsally depressed portion of pars distalis of penis, its tip slightly extending the distal margin (Fig. 14) of the ventral plate. Glans composed of two-thirds of prickly funnel and capsula externa near the base and one-third of stylus and capsula interna (Figs 14, 16). Capsula externa and capsula interna cylindrical, and the inner side of capsula interna with dense cover of fur-like microtrichia (Fig. 16). Stylus with irregular shape, constricted apically, the inverted stylus with capsula interna sunken into the spiny funnel, and all parts mentioned above surrounded totally by the capsula externa (Fig. 16). Ventral plate with 18 large setae (Figs 13–15): four dorsal, eight lateral and six ventral.

**Female** (Figs 8, 10, 12, 18–19). In general appearance similar to the male; abdomen more rounded posteriorly (Figs 20, 23). Granulation and spination of body similar to the male (Figs 8, 23). Chelicerae not enlarged but of normal shape, with a slight difference in inner edges of the cheliceral finger (Fig. 10). Pseudonychium of legs IV in females reduced compared to that of male (Figs 11, 12). Femora of pedipalpi dorsally with a row of six setiferous tubercles. Tarsal formula (I–IV): 5(2)/11–12(3)/6/7.

***Ovipositor*** (Figs 18, 19). Ventral side with four, dorsal side with six setae.

##### Measurements.

Male holotype (female paratype): Body 3.53 (3.53) long, 2.29 (2.00) wide at the widest portion. Scutum 1.85 (1.64) long. Interocular mound 0.63 (0.57) long, 0.34 (0.34) wide, 0.24 (0.20) high, 0.13 (0.12) far from the anterior border of the scutum. Pedipalpal claw 0.39 (0.45) long. Penis 1.02 long. Measurements of left pedipalpus and legs as in Tables 1, 2.

**Table 1.** *Linzhiassamiamedogensis* sp. nov. Measurements of the pedipalp and legs of the male holotype (MHBU-Opi-24ZC011501), as length/width.

	Trochanter	Femur	Patella	Tibia	Metatarsus	Tarsus	Total
Pedipalp	0.47/0.22	1.09/0.32	0.78/0.24	0.67/0.29		0.58/0.28	3.59
Leg I	0.36/0.25	1.73/0.21	0.63/0.28	1.26/0.18	1.98/0.09	1.18/0.05	7.14
Leg II	0.39/0.30	3.22/0.20	0.94/0.29	2.72/0.16	3.24/0.10	2.52/0.07	13.03
Leg III	0.46/0.37	2.30/0.24	0.81/0.35	1.51/0.21	2.68/0.16	1.40/0.11	9.16
Leg IV	0.60/0.36	3.75/0.26	1.02/0.42	2.23/0.21	4.32/0.18	1.89/0.12	13.81

**Table 2.** *Linzhiassamiamedogensis* sp. nov. Measurements of the pedipalp and legs of the female paratype (MHBU-Opi-24ZC011503), as length/width.

	Trochanter	Femur	Patella	Tibia	Metatarsus	Tarsus	Total
Pedipalp	0.44/0.22	0.97/0.28	0.70/0.24	0.62/0.28		0.54/0.24	3.27
Leg I	0.45/0.28	2.45/0.16	0.72/0.25	2.35/0.15	2.83/0.08	2.08/0.08	10.88
Leg II	0.46/0.29	2.75/0.18	0.74/0.24	2.44/0.15	2.71/0.08	2.04/0.08	11.14
Leg III	0.42/0.35	2.03/0.23	0.58/0.31	1.32/0.22	2.21/0.13	1.21/0.11	7.77
Leg IV	0.54/0.33	3.09/0.23	0.78/0.32	1.92/0.23	3.45/0.14	1.52/0.08	11.30

##### Habitat.

The specimens were collected under stones and on the leaves of the shrubbery.

##### Distribution.

Known only from the type locality, the Medog County, Nyingchi City, Xizang Autonomous Region, China.

##### Variation.

Specimens examined included three males and two females, the number of tarsal segments on the second legs was not constant, which varied from eleven to fourteen segments. On the fourth leg, the number of tarsal segments varies from seven to eight. The number of tarsal segments on the second leg and third leg is constant, with five segments on the second and six segments on the third. Another variation is the number of setiferous tubercles on the pedipalpus trochanter. For example, the male holotype (MHBU-Opi-24ZC011501) has only one setiferous tubercle on the pedipalpus trochanter (Figs 5, 6), while the male paratype (MHBU-Opi-24ZC011601) has two setiferous tubercles (Fig. 1).

#### ﻿ Linzhiassamia zayuensis sp. nov.

Taxon classificationAnimaliaOpilionesAssamiidae

C33CCE25-0771-5751-B290-D3C18D0016EA

https://zoobank.org/65820C30-2533-44E6-A83F-94DABD451260

Figs 26–31 , 32–37 , 38–44 , 45–50 , 51–56 , 57–62 , 63–69 , 70–75

##### Type material.

***Holotype*** • male (MHBU-Opi-24ZC011801): China: Xizang, Nyingchi, Zayu County, 28°29'N, 97°30'E, alt. 1405 m, 13 July 2020, L.Y. Wang leg. ***Paratypes***: • one female (MHBU-Opi-24ZC011802), China: Xizang, Nyingchi, Zayu County, 28°77'N, 96°72'E, alt. 1945 m, 27 May 2019, H. Wang leg • one male and one female (MHBU-Opi-24ZC011803-04), China: Xizang, Nyingchi, Zayu County, 28°53'N, 96°99'E, alt. 1509 m, 11 May 2023, Y. M. Hou, Z. Y. Yang leg • one male (MHBU-Opi-24ZC011901), China: Xizang, Nyingchi, Lulang Town, 29°96'N, 94°82'E, alt. 2472 m, 21 May 2019, H. Wang leg • one female (MHBU-Opi-24ZC011902), China: Xizang, Nyingchi, Bome County, 30°10'N, 95°07'E, alt. 2037 m, 02 June 2022, B. Liu leg • one male and one female (MHBU-Opi-24ZC011903-04), China: Xizang, Nyingchi, Bome County, 30°04'N, 95°02'E, alt. 2051 m, 17 July 2020, L. Y. Wang, Y. M. Hou, leg.

##### Diagnosis.

The distal margin of the ventral plate is smooth and without any indentation (Figs 38, 63). Capsula externa cylindrical and capsula interna triangular, and the inner side of capsula interna with dense cover of fur-like microtrichia (Figs 41, 66). The ocularium has either a short spine or is unarmed, and there are two or three spines on the lateral anterior margin of the carapace (Figs 26, 32, 33, 45, 46, 48, 49, 51, 57, 58, 70, 71, 73–74).

##### Notes.

The external morphological differences of this species are significant. The interocular have either a short spine or are smooth, and at the lateral portion there are two or three spines on the anterior margin of the carapace. However, the seta-tipped tubercles on the opisthosomal region of scutum are relatively small, which allows for a preliminary differentiation from the other three species. Additionally, by examining the expanded structure of the genitalia together with external morphological characteristics, this species can be accurately distinguished from others.

##### Etymology.

The name of this species is derived from its collection locality in Zayu County, Nyingchi, Xizang.

##### Description.

**Figures 26–31.**
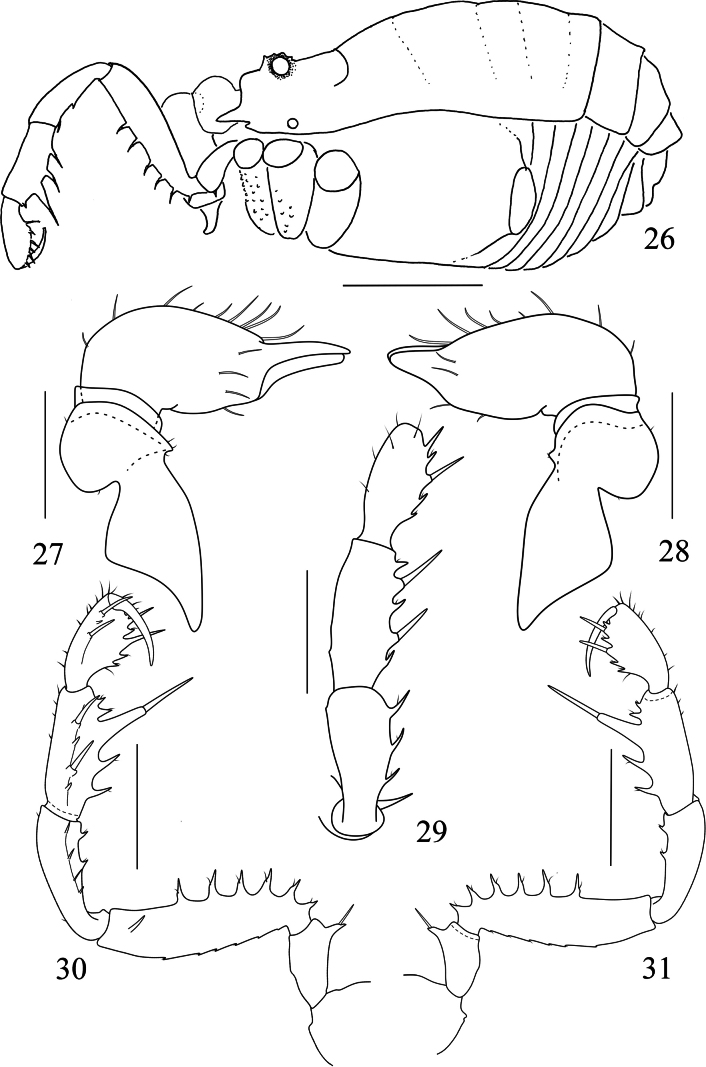
*Linzhiassamiazayuensis* sp. nov., male (**27–31** holotype), male (**26** paratype) **26** male body, lateral view **27** left chelicera of male, ental view **28** left chelicera of male, ectal view **29** left pedipalp of male, dorsal view **30** left pedipalp of male, ental view **31** left pedipalp of male, ectal view. Scale bars: 1 mm (**26**); 0.5 mm (**27–31**).

**Figures 32–37.**
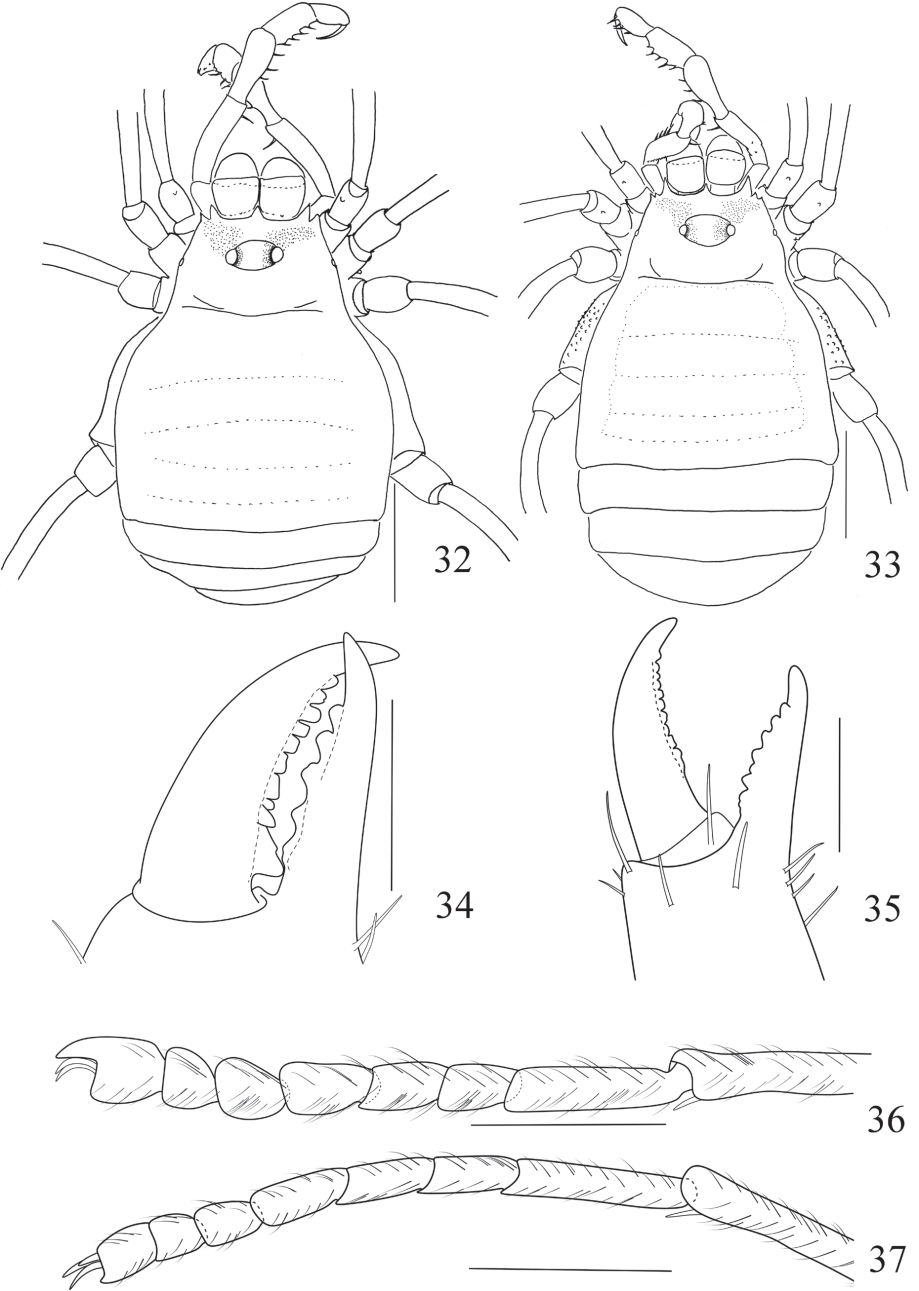
*Linzhiassamiazayuensis* sp. nov., male (**34, 36** holotype), male (**32** paratype), female (**33, 35, 37** paratype) **32** male body, dorsal view **33** female body, dorsal view **34** left cheliceral fingers of male, frontal view **35** left cheliceral fingers of female, frontal view **36** right tarsal claw IV of male, lateral view **37** right tarsal claw IV of female, lateral view. Scale bars: 1 mm (**32–33**); 0.5 mm (**36–37**); 0.25 mm (**34–35**).

**Male** (holotype and paratype). Habitus as in Figs 26, 32, 45–47. Coloration (Figs 45–47): entire body dorsally rusty yellow with brown patches; median area of prosoma with dark brown reticulations before and behind the interocular mound; anterior margin of prosoma have dark brown patches at the lateral portion; both lateral ridges of the prosomal and opisthosomal scutum with dark brown stripes; opisthosomal areas I–IV with dark brown patches, and there is a longitudinal dark brown stripe along the entire length; area V and free tergites each with a transverse dark band; The venter is the same color as the dorsum; chelicerae, pedipalps and legs rusty yellow, reticulated with light to dark brown.

***Dorsum*** (Figs 32, 45). Dorsal scutum pyriform in shape, widest portion of body at scutal area II. Anterior margin of carapace with three spines (two large spines and one small spine, with the middle one being the smallest) at the lateral portion and a single median spine; anterior margin of prosoma with two rows of small tubercles at the lateral portion. Ocularium oval, removed from the anterior border of scutum by 0.19 mm, and scattered with small tubercles. Opisthosomal region of scutum with five areas. Except for a few scattered small seta-tipped tubercles in areas II and III, opisthosomal areas I to IV are mostly smooth. Area V and all free tergites with a transverse row of seta-tipped tubercles.

**Figures 38–44.**
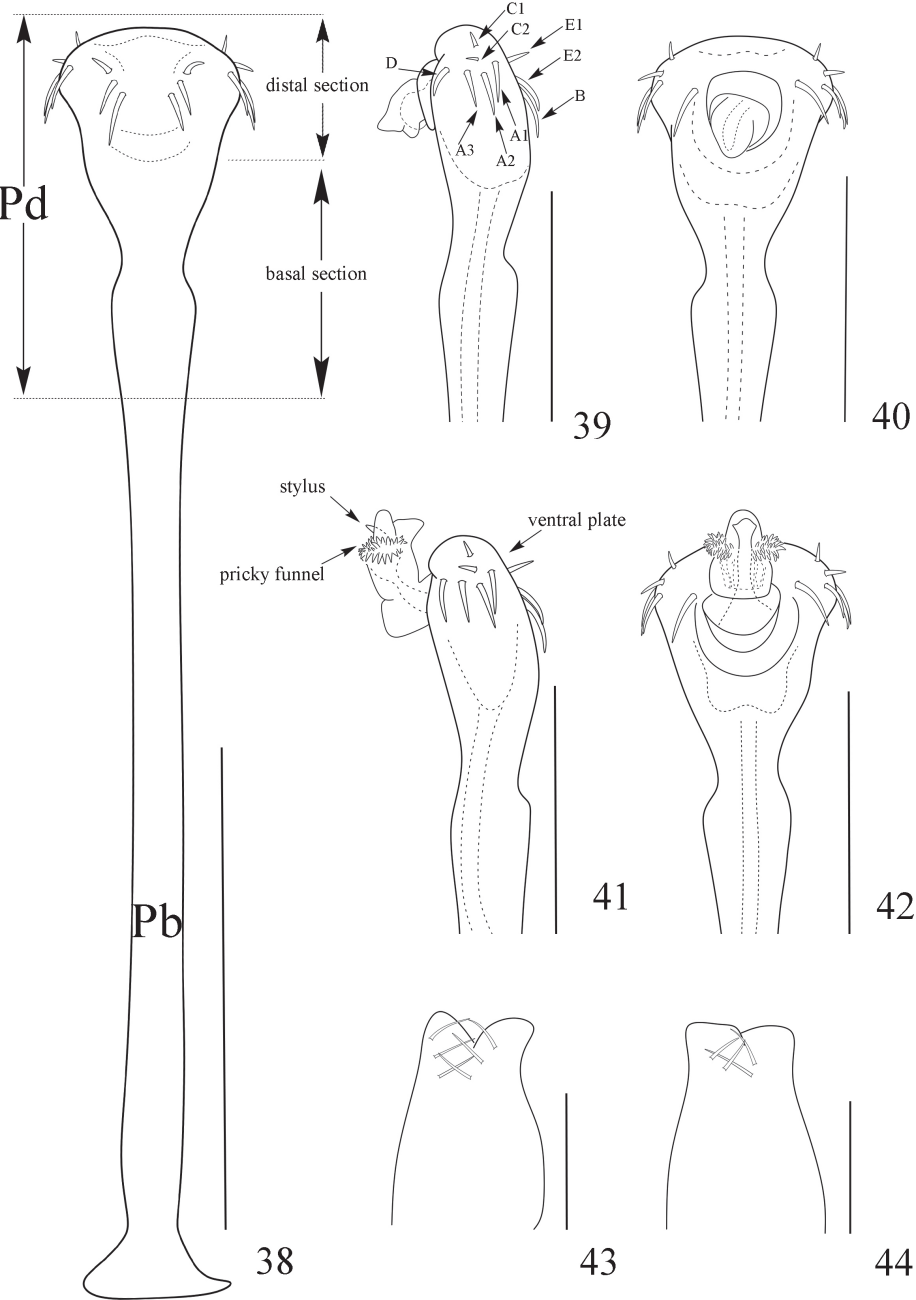
*Linzhiassamiazayuensis* sp. nov., genitalia of male holotype (**38–42**) and female paratype (**43, 44**) **38** penis, ventral view **39** distal part of penis, lateral view **40** same, dorsal view **41** distal part of penis (expanded), lateral view **42** same, dorsal view **43** ovipositor, dorsal view **44** same, ventral view. Pb, pars basalis, Pd pars distalis. Scale bars: 0.5 mm (**38**); 0.25 mm (**39–44**).

**Figures 45–50.**
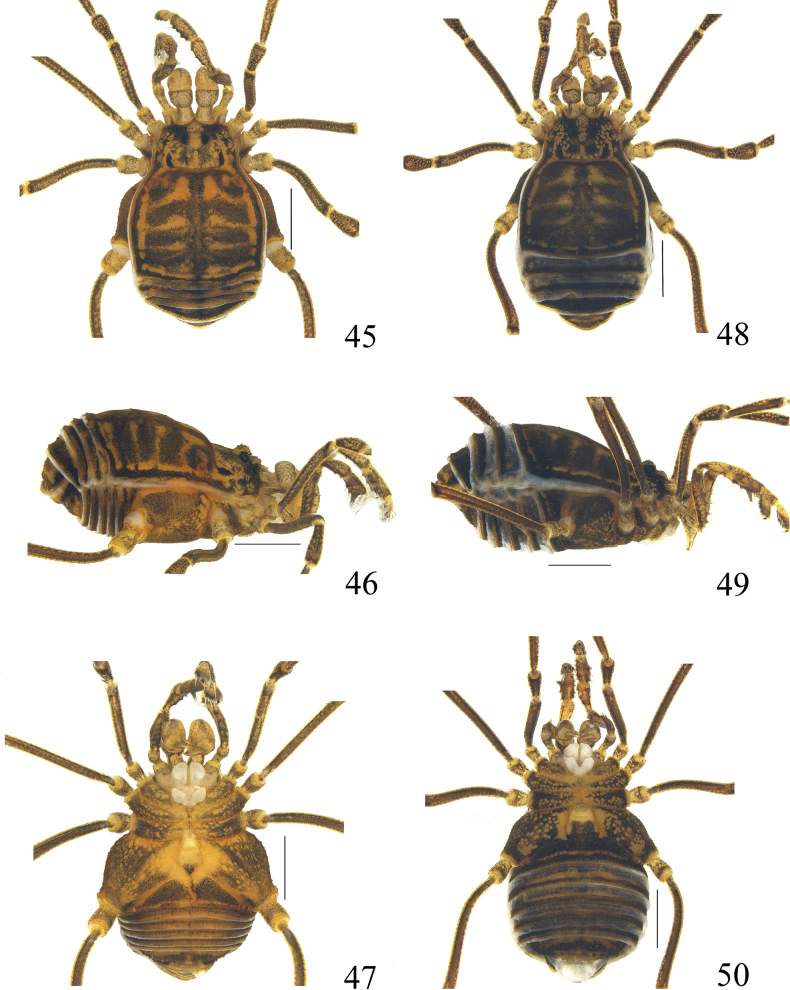
Photographs of male (**45–47** holotype) and female (**48–50** paratype) of *Linzhiassamiazayuensis* sp. nov. **45, 48** body and parts of appendages, dorsal view **46, 49** body and parts of appendages, lateral view **47, 50** body and parts of appendages, ventral view. Scale bars: 1 mm.

**Figures 51–56.**
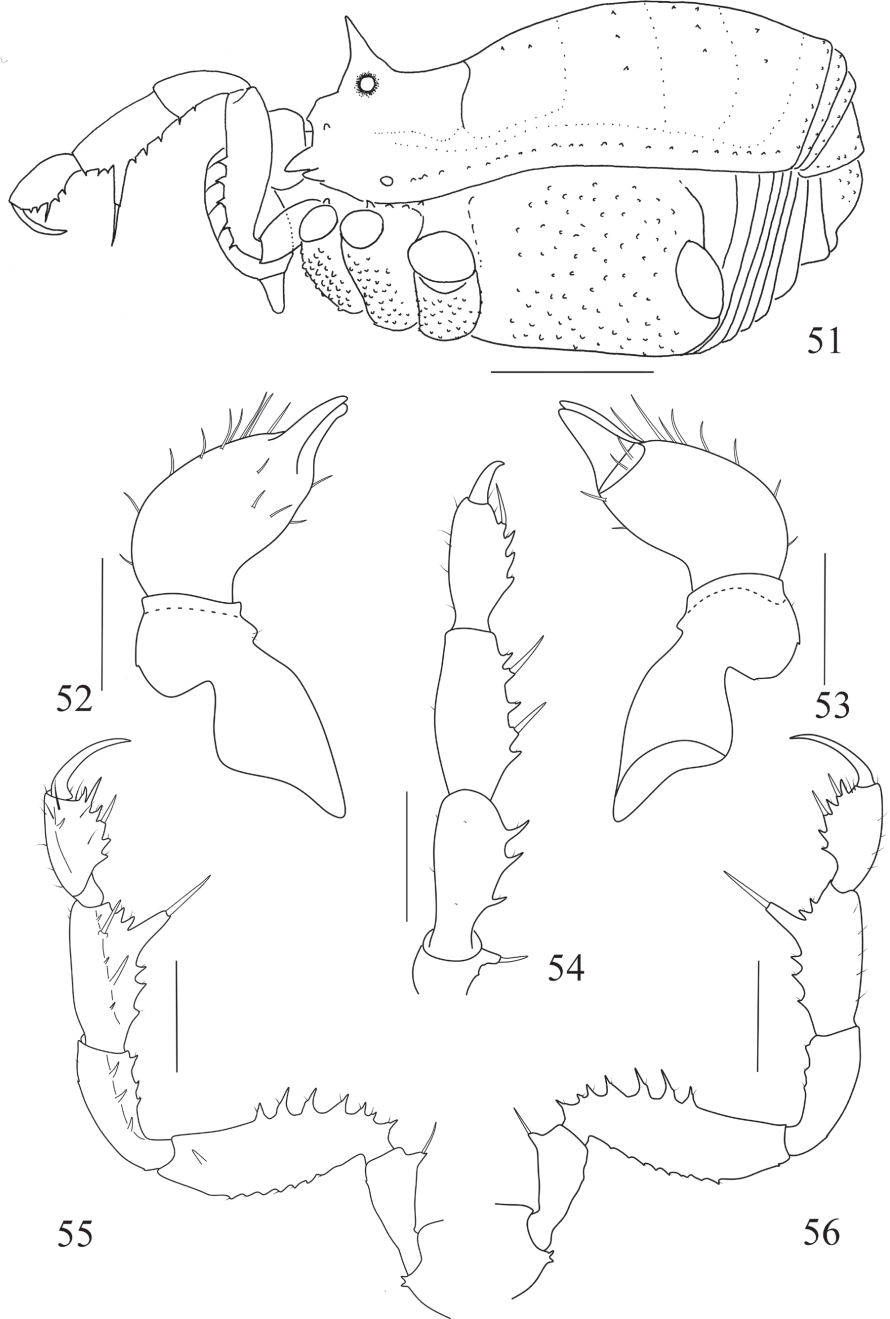
*Linzhiassamiazayuensis* sp. nov., male (**51–56** paratype) **51** male body, lateral view **52** left chelicera of male, ental view **53** left chelicera of male, ectal view **54** left pedipalp of male, dorsal view **55** left pedipalp of male, ental view **56** left pedipalp of male, ectal view. Scale bars: 1 mm (**51**); 0.5 mm (**52–56**).

**Figures 57–62.**
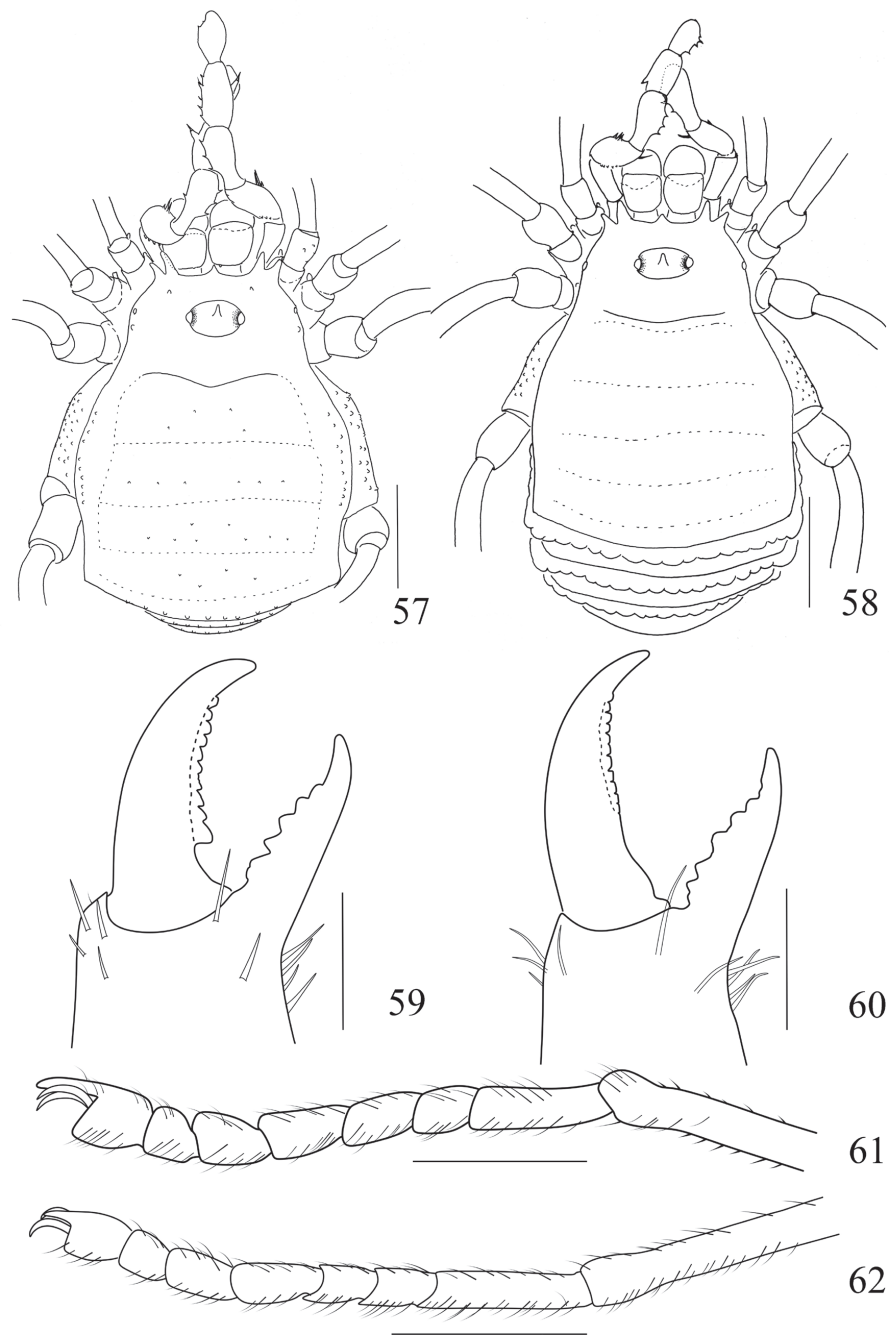
*Linzhiassamiazayuensis* sp. nov., male (**59, 61, 57** paratype), female (**58, 60, 62** paratype) **57** male body, dorsal view **58** female body, dorsal view **59** left cheliceral fingers of male, frontal view **60** left cheliceral fingers of female, frontal view **61** right tarsal claw IV of male, lateral view **62** right tarsal claw IV of female, lateral view. Scale bars: 1 mm (**57, 58**); 0.5 mm (**61–62**), 0.25 mm (**59–60**).

**Figures 63–69.**
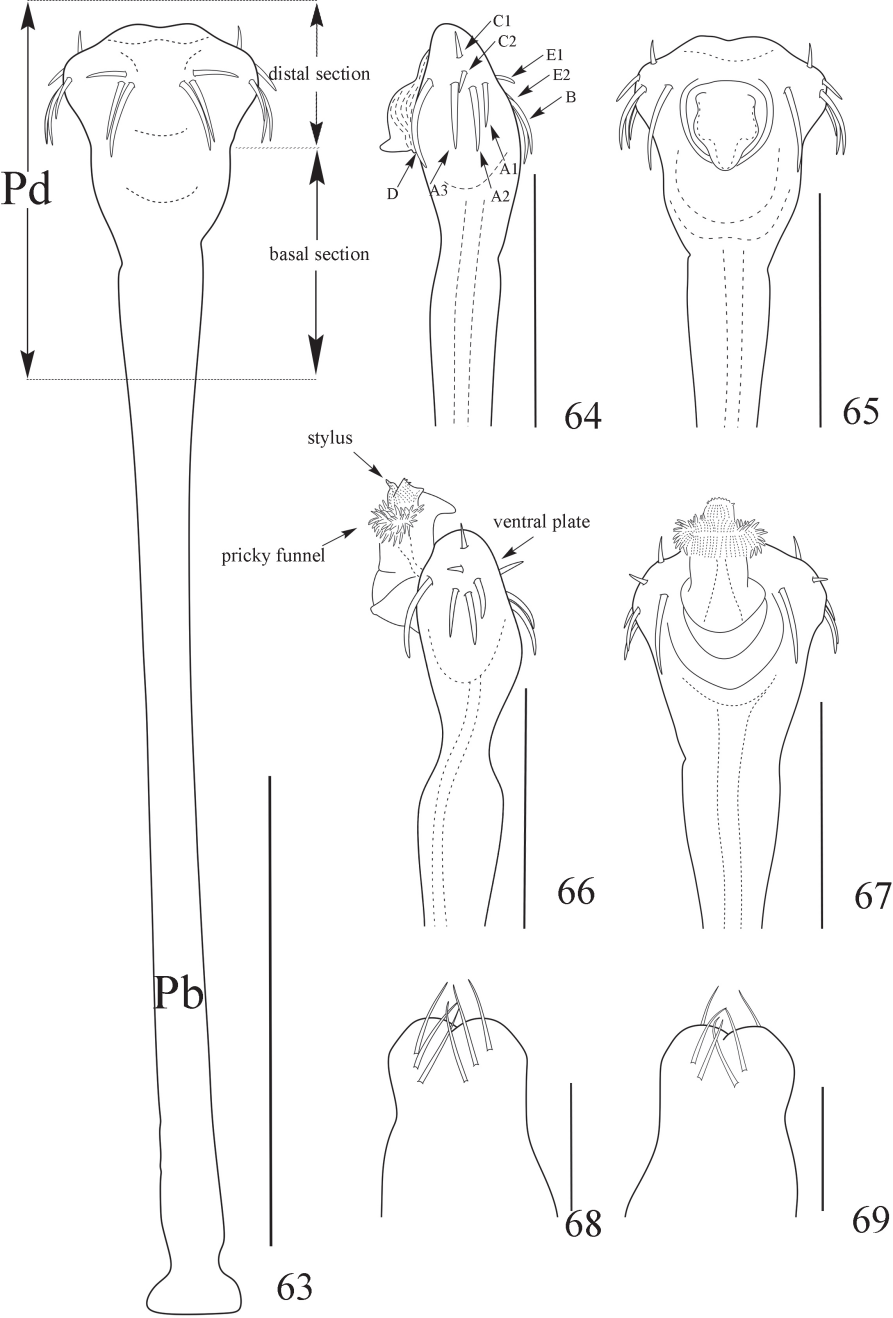
*Linzhiassamiazayuensis* sp. nov., genitalia of male paratype (**63–67**) and female paratype (**68–69**) **63** penis, ventral view **64** distal part of penis, lateral view **65** same, dorsal view **66** distal part of penis (expanded), lateral view **67** same, dorsal view **68** ovipositor, dorsal view **69** same, ventral view. Pb, pars basalis, Pd pars distalis. Scale bars: 0.5 mm (**63**); 0.25 mm (**64–69**).

**Figures 70–75.**
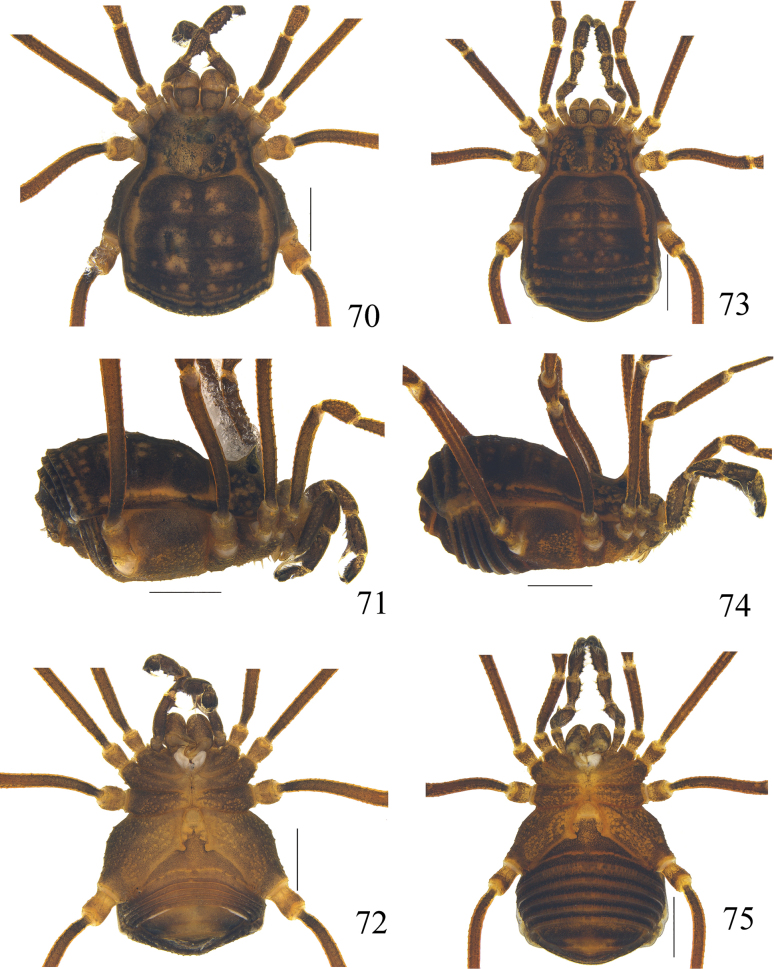
Photographs of male (**70–72** paratype) and female (**73–75** paratype) of *Linzhiassamiazayuensis* sp. nov. **70, 73** body and parts of appendages, dorsal view **71, 74** body and parts of appendages, lateral view **72, 75** body and parts of appendages, ventral view. Scale bars: 1 mm.

***Venter*** (Fig. 47). Surface of all coxae tuberculated. Coxa I with a row of four tubercles prolaterally, and two rows of tubercles on the surface. Coxa II with a row of marginal tubercles on the prolateral surface, and disto-dorsally with an enlarged tubercle. Coxa III with prolateral and retrolateral rows of tubercles. Coxa IV larger than others, prolaterally with a few scattered tubercles. Genital operculum with many hair-tipped granules. Free sternites with a row of minute tubercles. Spiracles concealed.

***Chelicera*** (Figs 27–28, 34). Basichelicerite elongate, dorsally with a slight bulla, without prominent armaments. Cheliceral hand unarmed, with sparse hairs only. Fingers relatively short, inner edges toothed as illustrated (Fig. 34): moveable finger with 10 teeth, the proximal one enlarged; fixed finger with five teeth, the proximal one diminished.

***Pedipalpus*** (Figs 29–31). Coxa dorsally with one small tubercle. Trochanter ventrally with one long distal setiferous tubercle. Femur compressed laterally, widest at the middle of its length, ventrally with a row of six homogeneous setiferous tubercles; dorsally with a row of six low conical tubercles along the entire length; on the medial distal side with one setiferous tubercle. Patella with two ventromesal setiferous tubercles and four ventroectal setiferous tubercles. Tibia ventromesally with two enlarged and two small setiferous tubercles; and ventroectally with one fairly enlarged and four setiferous tubercles. Tarsus with sparse hairs, ventromesally with two slightly enlarged and one small setiferous tubercle, and ventroectally with two slightly enlarged and five small setiferous tubercles. Tarsal claw slightly curved, shorter than tarsus.

***Legs*.** Slender and elongated. Trochanters I–IV with small hair-tipped granules on the ventral surface. All femora with hair-tipped granules, femora III and IV curved. Tarsi III–IV with a pseudonychium and two bare claws (Fig. 36). Tarsal formula (I–IV): 5(2)/9–10(3)/6/7. Distitarsus I two-jointed and II three-jointed. The remaining leg segments with hair-tipped granules.

***Penis*** (Figs 38–42). Truncus (pars basalis) slender, sides nearly parallel, then slightly enlarged (Fig. 38). Distal portion of penis (pars distalis) markedly enlarged: ventral plate nearly triangle, convex in dorsal view and concave in ventral view (Figs 39, 41), distal margin smooth and without any indentation (Figs 38, 40). Glans partially sunken into dorsal depressed portion of pars distalis and not extending the distal margin of the ventral plate (Fig. 39). The glans is composed of three-quarters by the prickly funnel and capsula externa near the base, and one-quarter by the stylus and capsula interna (Fig. 41). Capsula externa cylindrical and capsula interna triangular, and the inner side of capsula interna with dense cover of fur-like microtrichia. Stylus with irregular shape, constricted apically, the inverted stylus with capsula interna sunken into the spiny funnel, and all parts mentioned above surrounded totally by the capsula externa (Fig. 41). Ventral plate with 18 setae (Figs 38–40): two dorsal, 10 lateral and six ventral.

**Female** (Figs 33, 35, 37, 48–50). In general appearance similar to the male (Figs 33, 48–50). The chelicerae are not enlarged and have a normal shape, with a slight difference in the inner edges of the cheliceral fingers compared to the males. The movable finger with 11 teeth and the fixed finger with seven teeth, both more than in males (Fig. 35). Pseudonychium of legs IV in female reduced compared to that of male (Fig. 37). Femora of pedipalpi dorsally with a row of six setiferous tubercles. Tarsal formula (I–IV): 5(2)/9–10(3)/6/7.

***Ovipositor*** (Figs 43, 44). Ventral side with four, dorsal side with six setae.

##### Measurements.

Male holotype (female paratype): body 3.42 (3.88) long, 2.33 (2.30) wide at the widest portion. Scutum 2.60 (1.74) long. Interocular mound 0.50 (0.54) long, 0.30 (0.23) wide, 0.19 (0.13) high, 0.19 (0.26) far from the anterior border of the scutum. Pedipalpal claw 0.43 (0.33) long. Penis 1.34 long. Measurements of left pedipalpus and legs as in Tables 3, 4.

**Table 3.** *Linzhiassamiazayuensis* sp. nov. Measurements of the pedipalp and legs of the male holotype (MHBU-Opi-24ZC011801), as length/width.

	Trochanter	Femur	Patella	Tibia	Metatarsus	Tarsus	Total
Pedipalp	0.37/0.21	0.93/0.26	0.72/0.20	0.60/0.25		0.51/0.26	3.13
Leg I	0.31/0.23	1.32/0.18	0.59/0.24	1.06/0.17	1.49/0.07	0.71/0.07	5.48
Leg II	0.35/0.27	2.27/0.18	0.73/0.23	1.96/0.19	2.13/0.07	2.21/0.07	9.65
Leg III	0.43/0.34	1.69/0.19	0.62/0.31	1.22/0.24	1.91/0.12	1.14/0.08	7.01
Leg IV	0.52/0.36	2.41/0.19	0.79/0.36	1.74/0.26	2.78/0.16	1.54/0.10	9.78

**Table 4.** *Linzhiassamiazayuensis* sp. nov. Measurements of the pedipalp and legs of the female paratype (MHBU-Opi-24ZC011802), as length/width.1

	Trochanter	Femur	Patella	Tibia	Metatarsus	Tarsus	Total
Pedipalp	0.39/0.19	0.89/0.25	0.67/0.20	0.59/0.22		0.53/0.21	3.07
Leg I	0.29/0.21	1.30/0.19	0.58/0.20	0.98/0.17	1.40/0.07	1.02/0.06	5.57
Leg II	0.39/0.25	2.17/0.17	0.64/0.23	1.97/0.17	2.16/0.08	2.06/0.08	9.39
Leg III	0.37/0.27	1.71/0.20	0.57/0.26	1.11/0.21	1.86/0.08	1.19/0.07	6.81
Leg IV	0.43/0.26	2.27/0.22	0.66/0.27	1.55/0.21	2.82/0.09	1.52/0.07	9.25

##### Habitat.

These specimens were collected by sifting through the fallen leaves in the dark and humid undergrowth of the forest, as well as under stones and on the leaves of the shrubbery.

##### Distribution.

Known only from the type locality, the Zayu County, Bome County, and Lulang Town, Nyingchi City, Xizang Autonomous Region, China.

##### Variation.

Five male specimens were examined, displaying two distinct external morphologies, with a male paratype (MHBU-Opi-24ZC011901) chosen for discussion due to its differences from the male holotype (MHBU-Opi-24ZC011801). Compared to MHBU-Opi-24ZC011801, MHBU-Opi-24ZC011901 exhibits darker body coloration, there are no dark brown patches located on the sides of the anterior margin of the prosoma and larger dark brown patches on the opisthosomal areas I–IV (Figs 70–72). The carapace is shorter and rounder, with only two spines on each side of the anterior margin of the cephalothorax, ocularium armed with a conspicuous short median spine (Figs 51, 57, 70–71). Pedipalpus femur ventrally with a row of seven homogeneous setiferous tubercles, which has one more than MHBU-Opi-24ZC011801 (Figs 54–56). The chelicerae and inner edges of cheliceral finger show no significant differences (Figs 52, 53), the moveable finger with 10 teeth, fixed finger with six teeth (Fig. 59). There is no variation in pseudonychium of legs IV among male individuals. (Fig. 61).

Females also exhibit variation in morphological characteristics. Similar to the differences observed in males, females also display slight differences in body coloration (Figs 73–72). The number of spines on the anterior margin of carapace at the lateral portion from two to three, and the presence or absence of spines on the ocularium varies (Figs 73–75). Furthermore, the number of homogeneous setiferous tubercles on the pedipalpus femur ventrally ranges from six to seven. The chelicerae and inner edges of cheliceral finger show no significant differences, the moveable finger with 11 teeth, fixed finger with six teeth (Fig. 60). There is no variation in pseudonychium of legs IV and the ovipositor among females (Figs 62, 68, 69).

The observed external morphological differences initially led us to consider these as potentially separate species. However, upon dissecting the genitalia, we found remarkable similarities both before and after expansion, with only slight differences in the shapes of the stylus and prickly funnel (Figs 63–67). We speculate that these differences may be due to varying degrees of genitalia expansion. Despite the significant variations in external morphology, including coloration, interocular tubercles, pedipalp spines, and cheliceral teeth, our examination of the male genitalia did not reveal sufficient divergence to warrant the classification of distinct species at this time.

The Qinghai-Xizang Plateau is characterized by high altitude, thin atmospheric layers, and unique geographical and climatic conditions. These factors likely contribute to the observed morphological variations among specimens, particularly given the restricted gene flow between populations at different altitudes due to the limited dispersal capacity of harvestmen. While these variations may suggest the presence of more than one species, the current sample size and geographic coverage do not provide enough evidence to definitively separate these populations into distinct species. A more extensive collection of specimens from a broader range of localities is necessary before any formal taxonomic decisions can be made.

## ﻿Discussion

The phylogenetic analysis by Palmieri et al. (2023) serves as an excellent foundation for comprehending the primary lineages of Assamiidae, particularly as they largely align with geographic distributions. However, the non-Afrotropical part of that study omits Himalayan species and only lightly addresses the Indian Trionyxellinae, primarily focusing on insular species (Dampetrinae).

We may endeavor to categorize the existing diversity of continental Asian Assamiidae into coherent groups of species, disregarding the Roewerian subfamilies which rely on superficial traits. This way, evaluating the affinities of any genus within Assamiidae becomes challenging due to the often concise nature of descriptions. To accurately categorize subfamilies, we should reduce each to their type genus, and ideally, to their type species if we wish to designate a subfamily by name.

**Figure 76.**
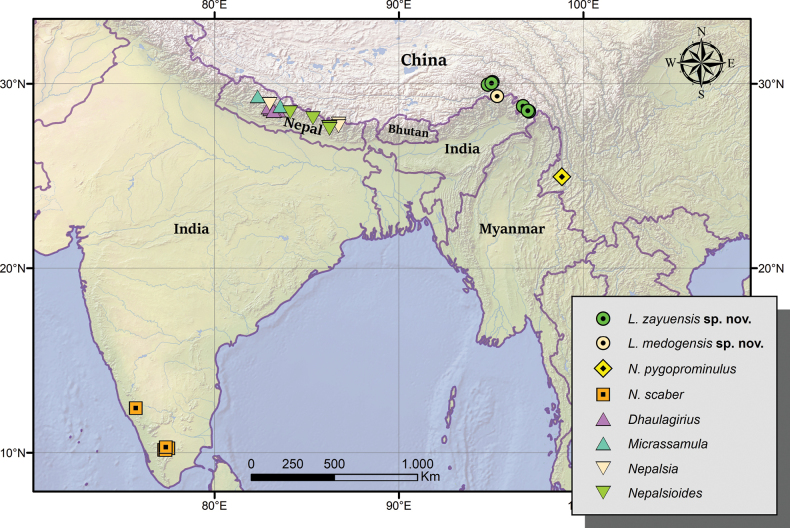
Eastern Himalaya and adjacent regions, showing the distribution of the species of *Linzhiassamia* and of probably related genera.

1. The assamiids possessing tarsi III–IV with a tarsal process (often called “pseudonychium”) were for a long time included together in the family Trionyxellidae Roewer, 1912, although there is no other evidence to assign these to a separate family. There are two subfamilies, the “true” Trionyxellinae and Mysoreinae, which should be restricted to southern India and Sri Lanka. This is the most poorly known group in Assamiidae, with no descriptions of genitalia.
2. Of the remnant pseudonychiate Asian assamiids, at least the species of *Paktongius* Suzuki, 1969 distributed in Laos and Thailand were said to possess a “gonyleptoid” condition: a lateral expansion of scutal areas III and IV and the hypertrophy of coxae IV (Klementz and Sharma 2023: 34). Some *Triaenopodium* Roewer, 1915 from Peninsular Malaysia, have similar genitalia and hints of this “gonyleptoid” condition (Suzuki 1976c: figs 11, 12). Another possible representative of this group is *Bandonaboninensis* Suzuki, 1974 from the Eastern Palearctic, with a male specimen from Yunnan depicted in Zhang et al. (2010: figs 1–16).
3. The Assamiinae sensu stricto having the distal part of the truncus shaped like an ice-cream scoop, with macrosetae elongated and prostrated. There are several examples from Nepal of this putative group, illustrated in Martens (1977), such as *Micrassamula* Martens, 1977 and *Nepalsioides* Martens, 1977 (Fig. 76). These “true” Assamiinae probably encompass species from Assam, West Bengal, Meghalaya, Arunachal Pradesh, Bangladesh, and Nepal.
4. There are also a few Assamiidae with strong sexual dimorphism on the chelicerae, and a peculiar genital configuration, which hail mostly from the highlands of Myanmar, Thailand, and Nepal. In *Euboeorix*, the distal truncus could be nicknamed “pancake bent”: flattened dorso-ventrally, widening laterally towards the distal end until it curves and terminates as a semicircle. Possible representatives of this group are *Aboriscus* Roewer, 1940, *Euboeorix* Roewer, 1912, and *Dhaulagirius* Martens, 1977.

*L.medogensis* sp. nov. and *L.zayuensis* sp. nov. exhibit pseudonychiate tarsal claws, prompting comparisons with Indian Trionyxellidae such as *Nilgirius*. However, a preliminary examination of the genitalia of *Nilgiriusscaber* Roewer, 1915 from southern India (Kury unpubl. data), reveals a markedly distinct genital structure. Our Tibetan species of *Linzhiassamia* also feature a pyriform distal truncus, which is not as depressed, with elongated macrosetae similar to those observed in *Dhaulagiriusaltitudinalis* Martens, 1977. However, the sexual dimorphism in the chelicerae is much more subtle.

While a comparison with *Dhaulagirius* is not out of the question, defining suprageneric groups for these highlanders remains a distant prospect. Knowledge of the highlands of the Eastern Himalaya remains fragmentary. There are no assamiids known from Bhutan, and the few species known from Arunachal Pradesh come from modest altitudes (up to 600 m) with only limited descriptions by Roewer.

## Supplementary Material

XML Treatment for Linzhiassamia

XML Treatment for Linzhiassamia medogensis

XML Treatment for Linzhiassamia zayuensis

## ﻿Additional information

### Author ORCIDs

Xiaoru Qi https://orcid.org/0009-0006-9458-2532

Adriano B. Kury https://orcid.org/0000-0002-8334-6204

Chao Zhang https://orcid.org/0000-0003-1702-1206

### Data availability

All of the data that support the findings of this study are available in the main text.
